# Establishment of a mouse model for ischaemic heart failure induced by coronary microembolization via left ventricular oil injection

**DOI:** 10.1113/EP092665

**Published:** 2025-10-19

**Authors:** Lang Pei, Yusheng Hong, Qiuyue Wu, Jianyi Lv, Changlong Li, Xueyun Huo, Xin Liu, Di Zhang, Meng Guo, Zhenwen Chen, Xiaoyan Du

**Affiliations:** ^1^ School of Basic Medical Science Capital Medical University Beijing Peoples’ Republic of China; ^2^ Laboratory for Clinical Medicine Capital Medical University Beijing Peoples’ Republic of China; ^3^ Beijing Key Laboratory of Cancer Invasion & Metastasis Research Beijing Peoples’ Republic of China; ^4^ Beijing Friendship Hospital Capital Medical University Beijing Peoples’ Republic of China; ^5^ SPF(Beijing) Biotechnology Co., Ltd Beijing Peoples’ Republic of China

**Keywords:** animal models, heart failure, microembolization

## Abstract

Over the past three decades, there has been a steady increase in clinical attention to ischaemic heart failure caused by coronary microembolization. Nonetheless, a suitable mouse model for studying this condition remains limited. In the present study, we developed a mouse model of coronary microembolization‐induced ischaemic heart failure by injecting corn oil into the left ventricle. After oil injection, the photoacoustic microscopy system identified an immediate obstruction of blood flow in the microvessels of the left ventricle. One day after injection of 10–60 µL oil, mice exhibited a significant decrease in left ventricular ejection fraction and fractional shortening, along with an increase in left ventricular volume during systole and elevated serum creatine kinase MB levels. Oil injection also resulted in acute cardiomyocyte necrosis, apoptosis and inflammatory infiltration. After 1 month, the mouse model demonstrated a prolonged and dose‐dependent reduction in heart function, in addition to significant increases in expression of the heart failure markers BNP and MYH7 and areas of cardiac fibrosis. Correspondingly, RNA sequencing revealed 126 differentially expressed genes in the oil‐injected mice, which were enriched in pathways related to myocardial contraction, vasodilatation, myocardial fibrosis and chemotactic inflammation. Notably, treatment with nitroglycerin or sacubitril–valsartan was able to mitigate the decrease of heart function in the mouse model. In conclusion, we successfully established a coronary microembolization‐induced mouse model of ischaemic heart failure by left ventricular injection of oil, which exhibited high sensitivity to nitroglycerin and sacubitril–valsartan.

## Abstract (highlights)

- **What is the central question of this study?**
  We explored the feasibility of using corn oil to obstruct the coronary artery microcirculation, in order to establish a mouse model for ischaemic heart failure induced by coronary microembolization.
- **What is the main finding and its importance?**
  We successfully developed a mouse model of ischaemic heart failure induced by coronary microembolization through oil injection into left ventricle. The model was characterized by immediate microvascular obstruction, cardiomyocyte necrosis, apoptosis, inflammatory infiltration, ongoing scarring of heart tissues and a high sensitivity to nitroglycerin and sacubitril–valsartan. This model might serve as a valuable tool for investigating the pathogenetic mechanisms and treatment of coronary microembolization‐induced ischaemic heart failure.

## 1 INTRODUCTION

Heart failure is the leading cause of hospitalization for patients >65 years old, with a 5‐year survival rate of <50%. Although the causes of heart failure are diverse, ischaemic heart failure accounts for ∼50% of all types of heart failure (Liang et al., 2021). Over the past three decades, clinical investigations have revealed that debris and soluble factors originating from coronary atherosclerotic plaques, whether through natural processes or through interventions, can lead to coronary microembolization (Frink et al., 1988; Kleinbongard & Heusch, 2022; Kolte et al., 2021; Puymirat et al., 2017). This, in turn, triggers coronary microvascular dysfunction and the formation of patchy microinfarction with inflammation, which is thought to be an essential cause of myocardial contractile dysfunction and ischaemic heart failure (Rush et al., 2021). Nonetheless, research on coronary microembolization‐induced ischaemic heart failure is comparatively limited.

One possible reason for this is the shortage of a suitable animal model for studying coronary microembolization‐induced ischaemic heart failure. The ​left anterior descending coronary artery (LAD) ligation​ model effectively mimics ​epicardial coronary artery occlusion​ and the resulting ​myocardial infarction (Gistera et al., 2022; Shamsuzzaman et al., 2024), but ​fails to model​ coronary microvascular obstruction‐induced patchy microinfarction adequately. The western diet‐fed transgenic mouse (*Apoe* ^−/−^, *Ldlr* ^−/−^ or SR‐BI^∆CT/∆CT^/*Ldlr* ^−/−^) model replicates ​atherosclerosis‐induced myocardial infarction in humans, but shows ​heterogeneous coronary pathology, affecting both epicardial arteries and microvasculature. Critically, stroke occurs in 38% of mice, introducing ​significant confounding effects​ for cardiac‐focused studies (Gistera et al., 2022; Shamsuzzaman et al., 2024). Supraphysiological treatment with the β‐adrenergic receptor agonist isoprenaline can induce myocardial infarction primarily through mechanisms involving arterial hypotension and myocardial hyperactivity, leading to cardiomyocyte hypoxia and necrosis. However, isoprenaline also generates free radicals that cause direct, persistent mitochondrial damage and cardiotoxicity (Allawadhi et al., 2018). Consequently, this model does not accurately simulate the pathophysiology of myocardial infarction driven by coronary artery ischaemia. ​In contrast, in sheep, dogs and pigs, the use of polymer microspheres with varying diameters for intracoronary injection to induce microembolization has been a common practice, but the high cost of these animals must be taken into consideration (Carlsson et al., 2009; Dispersyn et al., 2002; Dorge et al., 2002; Eng et al., 1984; Grund et al., 1999; Saeed et al., 2013). In mice, polymer microspheres have also been used to induce microembolization through left ventricular injection. However, it is important to note that the microspheres have been found in other organs, such as brain and kidney, leading to disorganization and focal vacuole‐like alterations (Cao et al., 2016; Grimm et al., 1998). Recently, autologous thrombotic particles were used to induce microembolization in mice by left ventricular injection. Nevertheless, acute inflammatory infiltration and chronic pathogenic changes were not shown (Li et al., 2024). In light of this, it is crucial to find a proper material to induce an experimental mouse model that mimics coronary microembolization‐induced ischaemic heart failure, aiming for a close resemblance to clinical pathophysiological processes, ease of manipulation, time efficiency and high survival rates.

Plant oils, such as corn oil, are insoluble in blood, enabling appropriately sized droplets of these oils to obstruct blood vessels. Meanwhile, these oils can be emulsified and effectively cleared from peripheral tissues, in contrast to polymer microspheres and neutral fat isolated from animal adipose tissue (Takada et al., 2015; Vilaro & Llobera, 1988). Considering these properties, here we focus on exploring the feasibility of using corn oil to obstruct coronary artery microcirculation, in order to establish a mouse model for ischaemic heart failure induced by coronary microembolization. Furthermore, we seek to analyse the expression profiles of our model and to assess its drug sensitivity.

## 2 MATERIALS AND METHODS

### 2.1 Ethical approval

The experimental procedures and animal handling were strictly in line with the guidelines set by the Animal Experiments and Experimental Animals Management Committee of Capital Medical University. The research protocol underwent comprehensive evaluation and received approval from the Animal Experiments and Experimental Animal Welfare Committee at Capital Medical University (permit number AEEI‐2021‐152).

### 2.2 Animals

Eight‐week‐old male C57BL/6 mice (Beijing Vital Laboratory Animal Technology, China) were maintained in laboratory animal facilities of Capital Medical University in standard laboratory conditions, including a humidity range of 40%–65%, a temperature of 22°C ± 4°C and a 12 h–12 h light–dark cycle. The mice had free access to food and water throughout the study. The animals were killed by carbon dioxide (CO_2_) inhalation.

### 2.3 Left ventricular injection of oil

Mice were divided into six groups at random, with each group receiving a total volume of 80 µL of corn oil–normal saline suspension containing varying amounts of oil (0, 10, 20, 40, 60 or 80 µL). General aneesthesia was induced by intraperitoneal injection of 1.25% tribromoethanol (T48402, Sigma‐Aldrich, USA) solution at a dose of 200 mg/kg body weight. With the mice under general anaesthesia, 80 µL of suspension was drawn into a microsyringe. The apical projection area of the apex of the heart was identified by palpating the most intensely beating area of the xiphoid cartilage region with an index finger. The 27‐gauge needle was then carefully inserted perpendicularly to the sternum at this projection site. Bright red oxygenated blood spontaneously pumped into the needle hub, indicating the correct positioning of the needle. (If no blood or deep red blood appeared, the needle was withdrawn, and the apical projection area of left ventricle would be reidentified.) The suspension was administered slowly, after which the syringe was gently removed. Because of the natural elasticity of the heart wall, compression was applied for 1 min to stop any bleeding. After injection, mice were placed on a warming pad and monitored for respiration, reflex responses and thermoregulation until full recovery. Injection sites were inspected for haemorrhage or signs of infection. Body weight, food and water intake, wound healing and gait/posture were assessed daily after the injection. In accordance with the guidelines set by the Animal Experiments and Experimental Animals Management Committee of Capital Medical University, buprenorphine (0.1 mg/kg) was administered intraperitoneally twice daily on the day of injection for postoperative analgesia. If the mice subsequently exhibited pain behaviours corresponding to USDA Level D or higher, postoperative analgesia was continued.

### 2.4 Blood flow analysis of coronary arteries by photoacoustic microscopy

The photoacoustic microscopy (PAM) system Insight‐RSPAM1 (INNO LASER, China) was used to analyse blood flow in mice, as previously reported (Ren et al., 2024). Ten minutes or 1 month after oil injection, thoracotomy was performed under general anaesthesia. Imaging of blood flow in the coronary arteries was accomplished using a wavelength of 532 nm at a repetition rate of 10 kHz with a pulse width of 7 ns. The pulse laser energy was set to 800 nJ per pulse, and the scanning area at the anterior wall region of left ventricular was 5 mm × 5 mm. The photoacoustic signals generated by laser irradiation of the heart were received by a 50 MHz ultrasonic sensor. By selecting the isosbestic wavelengths for haemoglobin, the blood vessel flow could be normalized by the amplitude of photoacoustic signals of haemoglobin. The blood flow speed was estimated based on photoacoustic Doppler bandwidth broadening, determined by the standard deviation of 16 sequential A‐line scans (16 laser pulse illumination and 16 photoacoustic signals were performed at each image pixel). The acquired images were analysed quantitatively using ImageJ (National Institutes of Health, USA). Signal intensities within regions of interest were measured and normalized to background values. For thresholding, bright areas with distinct vascular structures were selected as statistical regions of interest. The areas of specific regions were quantified statistically across all samples. Thresholding and particle analysis tools were applied to ensure consistent segmentation.

### 2.5 Echocardiography

General anaethesia of the mice was maintained by inhalation of 1.5%–2.0% isoflurane on an insulated table at 37°C. Imaging was performed using a 40 MHz probe (MS‐550D, Visual Sonics, Canada) within a Vevo 2100 machine (FUJIFILM Visual Sonics, Canada) to evaluate parameters such as left ventricular anterior wall thickness, posterior wall thickness and internal diameters at end‐diastole and end‐systole. Subsequently, the left ventricular ejection fraction (EF), fractional shortening (FS) and left ventricular volumes were calculated.

### 2.6 Histological examination

Heart tissue was isolated, fixed in 4% polyoxymethylene overnight, embedded in paraffin and sectioned into 6‐µm‐thick slices, which were mounted on slides. The slides were then stained with Haematoxylin and Eosin or Masson's Trichrome and scanned under a Pannoramic scan (3D HISTECH, Hungary) to analyse cardiac morphology and fibrosis.

### 2.7 Terminal deoxynucleotidyl transferase‐mediated dUTP nick end labelling

 Terminal deoxynucleotidyl transferase‐mediated dUTP nick end labelling (TUNEL) was done with the TUNEL BrightRed Apoptosis Detection Kit (Vazyme, China) according to the manufacturer's instructions. Briefly, after a 30 min exposure to proteinase K solution, the slides were treated with 50 µL TdT plus 450 µL of fluorescein‐labelled dUTP solution at 37°C for 60 min. Next, 50 µL TUNEL reaction mix was added to the specimen, a coverslip was placed on top, and the reaction took place in a dark wet box for 60 min at 37°C. Apoptotic cells were photographed and analysed under a Leica SP8 laser scanning confocal microscope (Leica, Germany).

### 2.8 Quantitative RT‐PCR (qRT‐PCR)

Total RNA was extracted using TRIzol (Vazyme). Reverse transcription of cDNA was done using the FastQuant RT Kit (Tiangen, China). Messenger RNA levels were quantified by qRT‐PCR using CFX96″Optics Module (Bio‐Rad, USA) and QuantiTect SYBR Green PCR kits (Tiangen). Expression levels were calculated using the 2^−ΔΔ^ *^Ct^* method. Primers are listed in Table A1.

### 2.9 Enzyme‐linked immunosorbent assay

Serum creatine kinase MB (CK‐MB) levels were measured with the Mouse Creatine Kinase MB Isoenzyme ELISA Kit (Fine Biotech, China) according to the manufacturer's instructions. Briefly, 100 µL of standard or diluted serum (at a ratio of 1:4) was added to the wells, followed by incubation at 37°C for 90 min. Subsequently, the plate was washed three times before adding 100 µL of the biotin antibody working solution, then incubating at 37°C for 60 min. After another round of washing, 100 µL of horseradhish peroxidase–streptavidin working solution was added, then incubated at 37°C for 30 min, followed by five washes. Next, 90 µL of chromogenic substrate was added, with an additional incubation at 37°C for 10–20 min. Finally, 50 µL of reaction stop solution was added. The values of optical density at 450 nm were read immediately by Synergy H1 Microplate Reader (Biotek, USA) and the CK‐MB levels calculated.

### 2.10 Quantitative analysis of Evans Blue

Quantitative analysis of Evans Blue was used to determine blood perfusion of the liver, kidney, spleen and brain tissues (Tsurudome et al., 2022). Evans Blue solution (4 mL/kg, 2%) was injected into the mouse tail vein and allowed to circulate for 1 h. Subsequently, spleen, kidney and brain tissues were collected and incubated with dimethylformamide at 60°C for 24 h. After incubation, the samples were centrifuged at 1000*g* for 5 min to separate the supernatant. The absorbance of the supernatant at 620 nm was measured using a spectrophotometer (BIOTEK, USA). A standard curve was established by measuring the optical density values of Evans Blue standard samples at concentrations ranging from 0 to 200 ng/mL. The concentration of Evans Blue in the samples was then calculated.

### 2.11 RNA sequencing and differential expression analysis

Heart samples were collected and sent to Berry Genomics (China) to perform RNA sequencing (RNA‐seq) using the Illumina NovaSeq 6000 platform. Clean data were generated after removal of low‐quality base or high‐percentage N base sequences and adaptors, then aligned to the reference genome using HISAT2. The *p*‐values were adjusted using the false discovery rate correction method developed by Benjamini and Hochberg, and genes with adjusted *p*<0.05 and fold changes of ≥1.2 were assigned as differentially expressed genes (DEGs).

### 2.12 Drug treatment

Mice in the sacubitril–valsartan group were injected intraperitoneally with sacubitril–valsartan solution (60 mg/kg/day, Novartis, Switzerland) for 1 day or 1 month, 2 h following the 60 µL oil injection, whereas the control group was injected with the same amount of normal saline. Mice in the nitroglycerin group were injected intraperitoneally with nitroglycerin solution (25 mg/kg/day, Beijing Yimin, China) for 1 day or 1 month, 2 h after the 60 µL oil injection, whereas the control group was given the same amount of normal saline.

### 2.13 Statistical analysis

Statistical evaluations were conducted using GraphPad Prism v.8.3 software (GraphPad Software, USA), with Student's paired *t*‐test and ANOVA as the analytical methods. Tukey's *post hoc* test was carried out following the identification of a significant *F*‐ratio via ANOVA. Statistical significance was determined at the *p* < 0.05 threshold. In each experimental set‐up, the sample size per treatment group exceeded three, with each experiment being conducted a minimum of three times to ensure reliability. Data are presented as the mean values ± SD.

## 3 RESULTS

### 3.1 Acute coronary microembolization and ischaemic heart failure in the mouse model with left ventricular injection of oil

**FIGURE 1:**
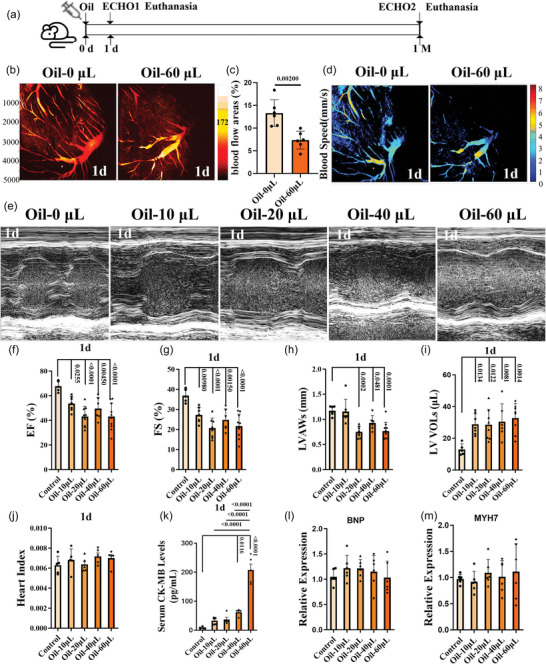
Acute coronary microembolization and ischemia heart failure in the mouse model 1 day after oil injection. The schematic diagram of the experimental protocol (a). The blood vessel flow (b), the blood flow areas (c) and the flow speed (d) of coronary arteries was shown by the photoacoustic microscopy (PAM) system. Echocardiography was performed on each group 1 day after injection (j), and left ventricular ejection fraction (EF), left ventricular short‐axis shortening rate (FS), systolic left ventricular anterior wall thickness (LVAWs) and systolic left ventricular volume (LV VOLs) were analyzed (e–i). After euthanasia, the heart index of mice (j) and the serum levels of creatine kinase MB (CK‐MB) (k) were detected. The expression levels of heart failure markers brain natriuretic peptide (BNP) and myosin heavy chain 7 (MYH7) were measured by qPCR (l–m). *n* = 5–10 mice (one RNA sample from each mouse) per group, ^*^ *p *< 0.05, ^**^ *p *< 0.01, ^***^ *p *< 0.001 and ^****^ *p *< 0.0001. Statistical analysis for panel c was performed using the Student's t‐test. For panels f–m, data were analyzed by one‐way ANOVA followed by Tukey's post hoc test. Results are presented as mean ± SD.

**FIGURE A1:**
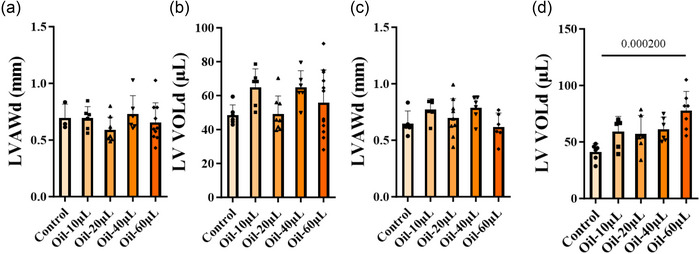
Diastolic left ventricular volumes in the mouse model with oil injection. Echocardiography was performed on each group 1 day after oil injection (a, b; *n* = 5–10 mice per group) and 1 month after injection (c, d; *n* = 5–8 mice), and the diastolic left ventricular anterior wall thickness (LVAWd) and diastolic left ventricular volumes (LV VOLd) were analysed. ^***^ *p *< 0.005. Data, presented as the mean ± SD, were analysed by one‐way ANOVA with Tukey's *post hoc* test.

To address whether left ventricular injection of oil could result in mouse heart dysfunction by damaging the local coronary blood supply, we injected 0, 10, 20, 40, 60 or 80 µL corn oil into the mouse left ventricle (Figure 1a). All mice survived after injection throughout the experimental period, except for those in the 80 µL group, which died within 2 min after injection. The PAM system identified an immediate and significant obstruction in blood flow of coronary microvessels in the left ventricle 10 min after injection of 60 µL of oil (Figure 1b–d). Echocardiographic examination revealed that left ventricular injection of 10–60 µL of oil led to a significant reduction in the percentage of EF and FS, in addition to left ventricular anterior wall systolic thickness, accompanied by a notable increase in left ventricular volume during systole 1 day after injection (Figure 1e–i). However, no significant changes were noted in the cardiac coefficients or in left ventricular anterior wall thickness and volumes during diastole (Figures 1j and A1a–b). To assess heart injury in the mouse model further, the myocardial necrosis marker creatine kinase MB (CK‐MB) and heart failure markers brain natriuretic peptide (BNP) and myosin heavy chain 7 (MYH7) were detected. The CK‐MB levels exhibited a dose‐dependent increase 1 day after injection (Figure 1k). The expressions of BNP and MYH7 were not altered (Figure 1l,m). Thus, the data indicate that left ventricular injection of oil in mice resulted in acute coronary microembolization and ischaemic heart failure.

### 3.2 Acute cardiomyocyte necrosis and apoptosis in the mouse model with left ventricular injection of oil

**FIGURE 2:**
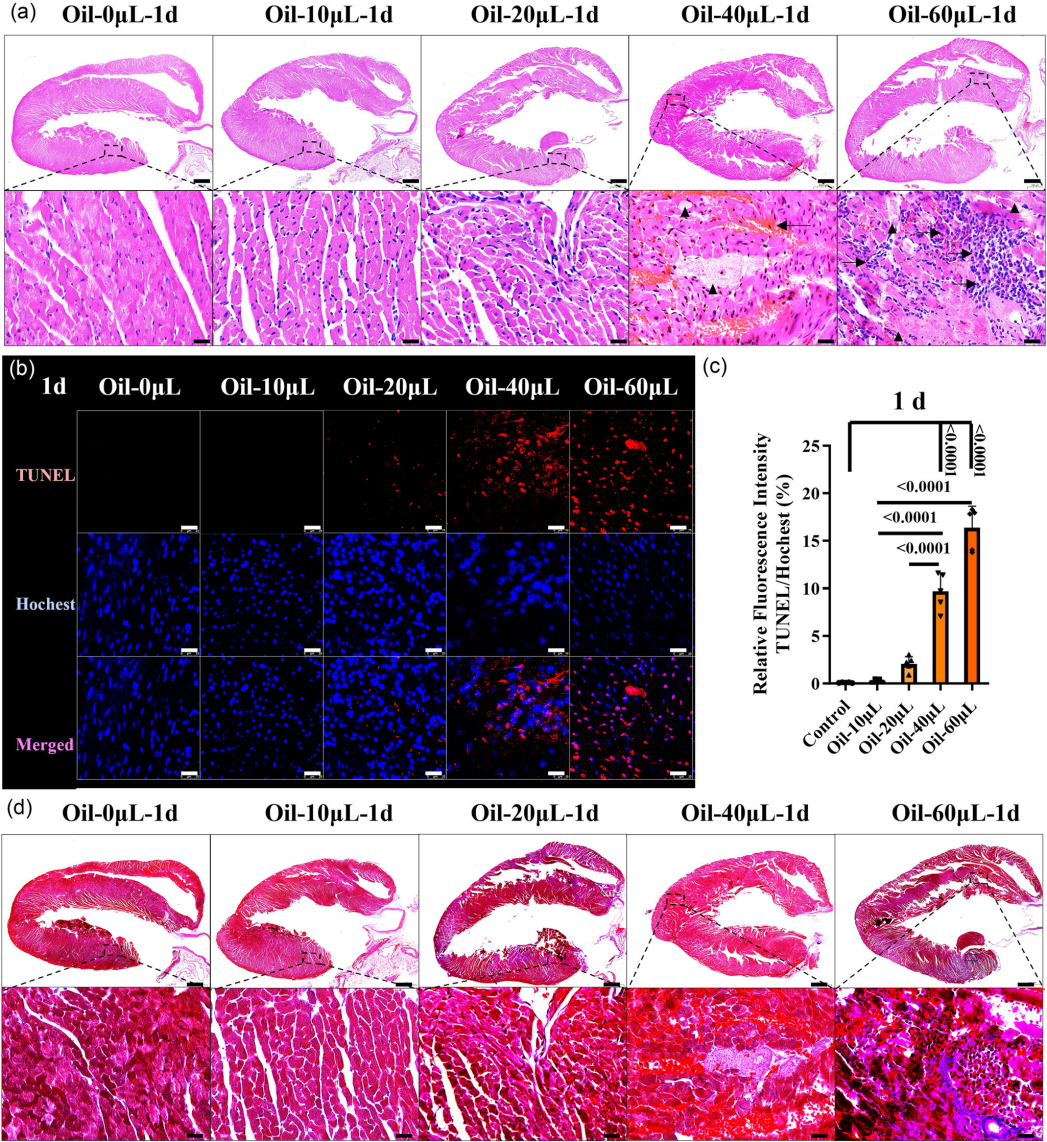
Acute cardiomyocyte necrosis and apoptosis in the mouse model 1 day (1d) after oil injection. (a) Haematoxylin and Eosin staining was carried out in all the groups 1 day after injection. Arrows indicate cardiomyocyte necrosis, and arrowheads indicate inflammatory infiltration. Scale bars: 500 µm in upper panels; 20 µm in lower panels. (b, c) Myocardial apoptosis was detected by TUNEL assay. Scale bars: 25 µm. (d) Masson staining was also performed. Scale bars: 500 µm in upper panels; 20 µm in lower panels. *n* = 5–10 mice (one slice from each mouse) per group; ^*^ *p *< 0.05, ^**^ *p *< 0.01 and ^****^ *p *< 0.0001. Statistical analysis for (c) was performed using one‐way ANOVA followed by Tukey's *post hoc* test. Results are presented as the mean ± SD.

**FIGURE A2:**
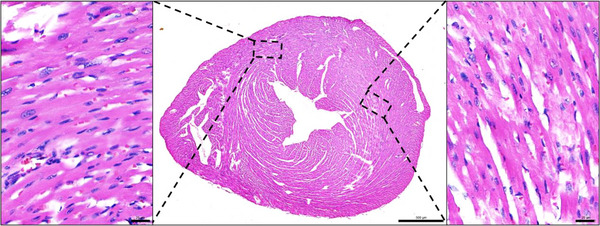
Disruption of the left ventricular wall in the mouse model with injection of 80 µL oil. After sudden death of mice following the injection of 80 µL oil, Haematoxylin and Eosin staining was promptly carried out. Scale bars: 500 µm (low magnification); 20 µm (high magnification). *n* = 4 (one slice analyzed per mouse).

**FIGURE A3:**
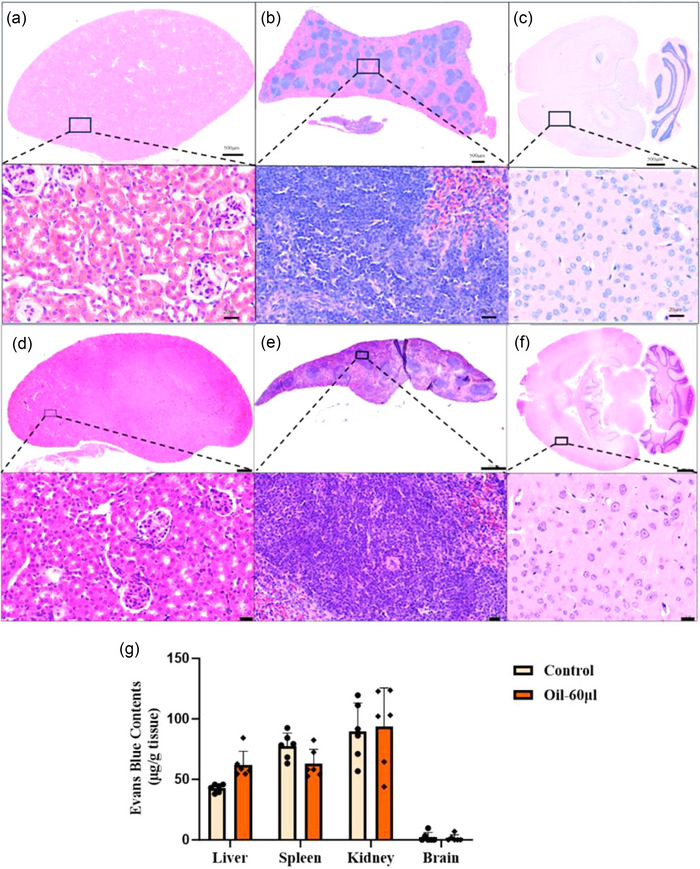
Morphological analysis and blood perfusion of the kidney, spleen and brain in the mouse model with injection of 60 µL oil. Haematoxylin and Eosin staining of kidney (a, d), spleen (b, e) and brain (c, f) was carried out in mice 1 day (a–c) and 1 month (d–f) after injection of 60 µL oil. Scale bars: 500 µm (kidney and spleen; upper panels); 1 mm (brain, upper panel); 20 µm (lower panels). *n* = 6 mice per group. (g) Impairment of the blood supply of kidney, spleen and brain was measured by Evans Blue quantification analysis 1 day after injection of 60 µL oil. *n* = 6 mice. Statistical analysis for (g) was performed using Student's *t*‐test. Results are presented as the mean ± SD.

To analyse the acute histological changes in heart after oil injection, Haematoxylin and Eosin, TUNEL and Masson's Trichrome staining were conducted. Coagulation necrosis of cardiomyocytes, haemorrhage and neutrophil infiltration were observed in the 60 µL group (Figure 2a), revealing that acute focal myocardial ischaemia happened. Autopsy revealed myocardial rupture in the 80 µL group (Figure A2a). In addition, the numbers of TUNEL‐positive cardiomyocytes in the left ventricular posterior walls near the atrioventricular septum showed a dose‐dependent increase 1 day after injection (Figure 2b, c). Cardiac fibrosis was absent (Figure 2d). Therefore, the data indicate that left ventricular injection of oil caused myocardial necrosis, apoptosis and inflammatory infiltration 1 day after injection. These acute morphological changes, which are highly comparable to those in coronary microembolization‐induced microinfarction (Kleinbongard & Heusch, 2022), appeared to contribute to the rapid decline in heart function. In addition, the impairment of morphology and blood supply of arterial organs such as kidney, spleen and brain were also assessed by Haematoxylin and Eosin staining and Evans Blue quantification, and no significant alterations were found in the oil‐injected groups (Figure A3a‐c,g).

### 3.3 Prolonged heart failure in the mouse model with left ventricular injection of oil

**FIGURE 3:**
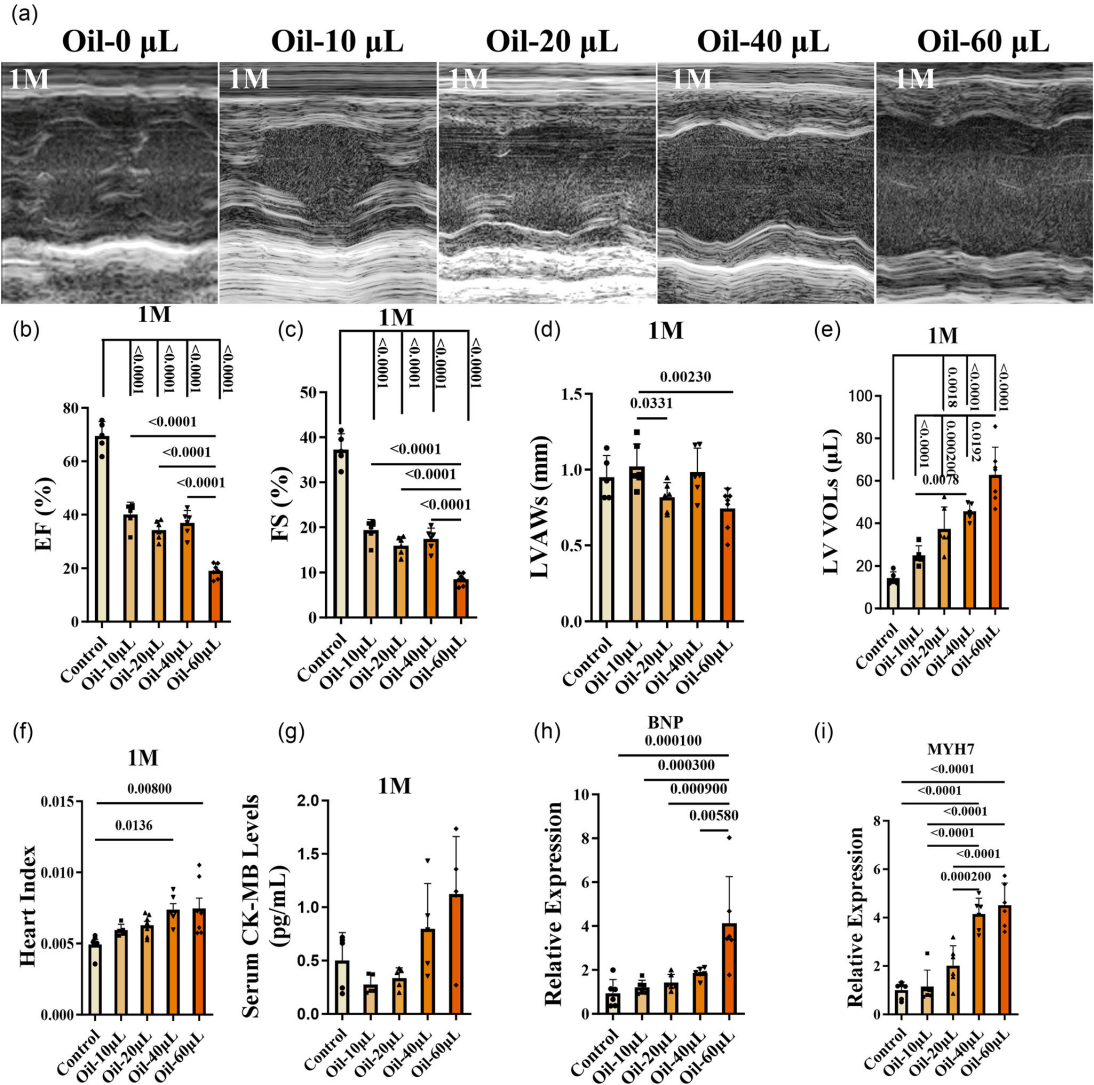
Prolonged heart failure in the mouse model 1 month after oil injection. (a) Mice were injected with 80 µL of oil–saline suspension containing 0, 10, 20, 40, 60 or 80 µL oil, respectively, and echocardiography was performed on each group 1 month after injection. (b–e) EF (b), FS (c), LVAWs (d), and LV VOLs (e) were analysed. (f, g) Post mortem, the heart index of mice (f) and the serum levels of CK‐MB (g) were detected. (h, i) Expression levels of heart failure markers BNP (h) and MYH7 (i) were measured by qPCR. *n* = 5–8 mice per group; ^*^ *p *< 0.05, ^**^ *p *< 0.01, ^***^ *p *< 0.001 and ^****^ *p *< 0.0001. For (b–i), data were analysed by one‐way ANOVA followed by Tukey's *post hoc* test. Results are presented as the mean ± SD.

**FIGURE A4:**
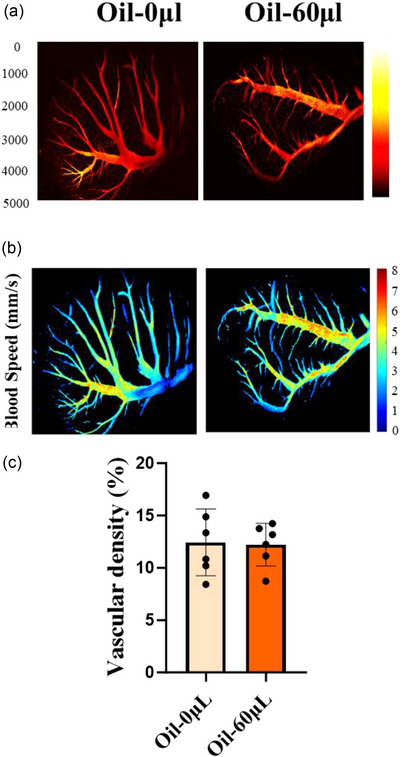
Blood flow in coronary microvessels of the left ventricle in the mouse model 1 month after oil injection. The blood vessel flow (a) and flow speed (b) of coronary arteries and blood flow areas (c) were assessed 1 month after injection of 60 µL oil using the photoacoustic microscopy (PAM) system. *n* = 5 mice per group. Statistical analysis in (c) was performed using Student's *t*‐test. Shown are averages with SD.

To evaluate the prolonged effects of left ventricular injection of oil further, we assessed blood flow and heart function 1 month after injection. Using the PAM system, we observed a recovery of blood flow in the coronary microvessels of the left ventricle when compared with measurements taken 1 day after injection (Figure A4a–c). However, heart systolic function deteriorated in a dose‐dependent manner in all oil‐injected groups, with left ventricular dilatation during systole (Figure 3a–e). The diastolic left ventricular volumes also rose in the 60 µL group (Figure A1c, d), and cardiac coefficients increased in the 40 and 60 µL groups (Figure 3f). But serum CK‐MB levels returned to levels comparable to the control group (Figure 3g). The expressions of BNP and MYH7 were higher than those of the control group (Figure 3h,i). Thus, the data indicate that left ventricular injection of oil resulted in prolonged and dose‐dependent heart failure in mice.

### 3.4 Cardiac fibrosis in the mouse model with left ventricular injection of oil

**FIGURE 4:**
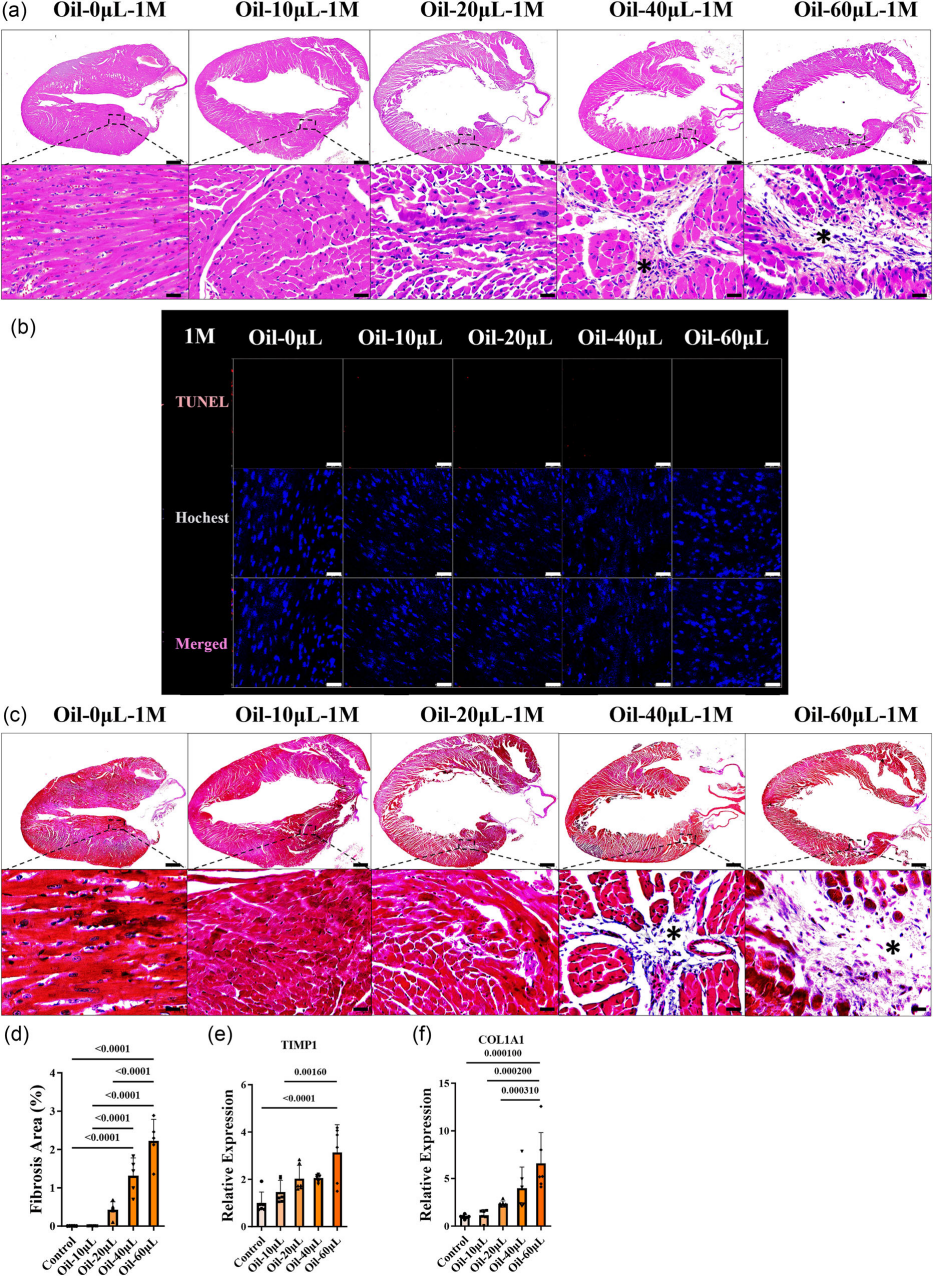
Heart fibrosis in the mouse model 1 month after oil injection. (a) Haematoxylin and Eosin staining was carried out in all the groups 1 month after injection. * indicates heart fibrosis. Scale bars: 500 µm in upper panels; 20 µm in lower panels. (b) Myocardial apoptosis was detected by TUNEL assay. Scale bars: 25 µm. (c, d) Masson staining (c) and the proportion of heart fibrosis (d) was assessed 1 month after oil injection. Scale bars: 500 µm in upper panels; 20 µm in lower panels. (e, f) The expression of fibrosis markers TIMP1 (e) and COL1A1 (f) was also detected by qPCR. *n* = 5–8 mice (one slice/RNA sample from each mouse) per group; ^*^ *p *< 0.05, ^**^ *p *< 0.01, ^***^ *p *< 0.001 and ^****^ *p *< 0.0001. For (d–f), data were analysed by one‐way ANOVA followed by Tukey's *post hoc* test. Results are presented as the mean ± SD.

To discover the prolonged histological alterations after oil injection, Haematoxylin and Eosin, TUNEL and Masson's Trichrome staining was also done 1 month after injection. Scar tissue was displayed in left ventricular posterior walls near the atrioventricular junction in the 40 and 60 µL groups (Figure 4a–c). The proportion of fibrotic area (Figure 4d) and the expression of cardiac fibrosis markers TIMP1 and COL1A1 increased in a dose‐dependent manner (Figure 4e,f). No apoptosis was observed in either group by TUNEL measurement (Figure 4b). Furthermore, impairment of the blood supply of the kidney, spleen and brain was also assessed by Haematoxylin and Eosin staining 1 month after left ventricular injection, and no significant changes were found (Figure A3d–f). These findings indicate that left ventricular injection of oil led to cardiac fibrosis in a dose‐dependent manner 1 month after injection.

### 3.5 The gene expression profiles in the mouse model with left ventricular injection of oil

**FIGURE 5:**
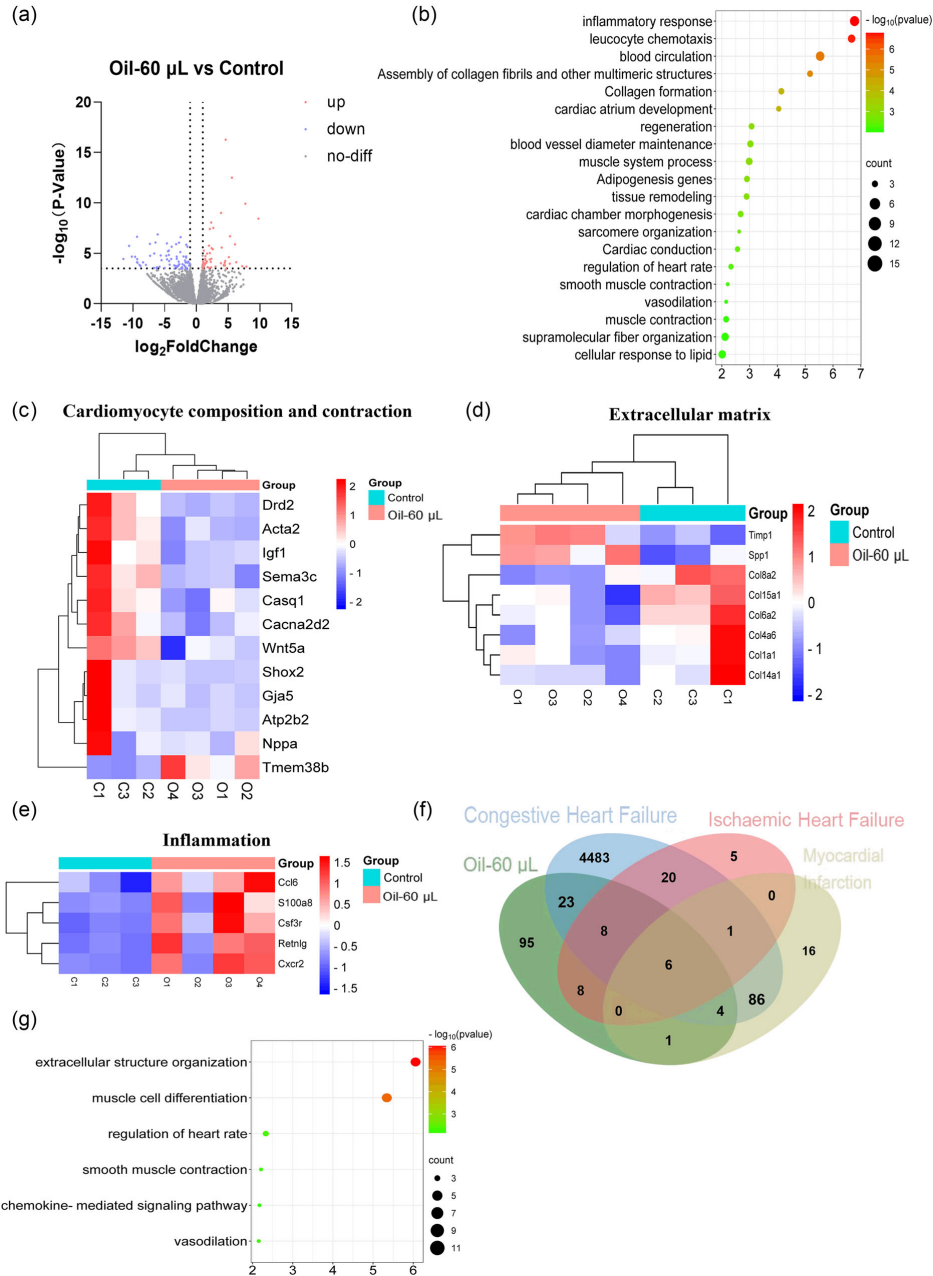
The gene expression profiles in the mouse model with left ventricular injection of oil. One day after injection with 80 µL of oil–saline suspension containing 0 or 60 µL oil, heart samples were collected and subjected to transcriptome sequencing (RNA‐seq). *n* = 3–4 (one RNA sample from each mouse) per group. (a, b) A volcano plot of the differentially expressed genes [DEGs; adjusted *p *< 0.05 and fold change (FC)≥1.2] (a) and bubble map depicting GO enrichment of DEGs (b) were generated. (c–e) Heat maps illustrating the expression of cardiomyocyte composition and contraction‐related genes (c), extracellular matrix‐related genes (d) and inflammation‐related genes (e) were also produced. (f) Venn diagram analysis was performed to compare the enriched biological processes among congestive heart failure patients (GSE1145 and GSE8331), ischaemic heart failure patients (GSE251971) and acute myocardial infarction patients (GSE76701) in the GEO database to our RNAseq data. (g) The intersecting categories among all the databases are presented.

To gain molecular insights into the heart failure mouse model, RNA‐seq was performed on the heart tissues of mice treated with 60 µL oil. One hundred and twenty‐six DEGs (fold changes > 1.2, adjusted *p* < 0.05) were present between the control and 60 µL groups. Fifty‐three genes were upregulated and 73 downregulated (Figure 5a, Supporting Information). GO enrichment analysis revealed significant biological processes, such as leucocyte chemotaxis, collagen formation, heart contraction and vasomotor tone (Figure 5b). Genes related to cardiomyocyte composition and contractility, such as *Igf1*, *Gja5* and *Acta2*, were reduced (Figure 5c). The expression of processes involved in the formation of connective tissue and collagen fibres was also changed (Figure 5d). Inflammation‐related genes, such as *Ccl6* and *S100a8*, were promoted in the 60 µL group (Figure 5e).

Moreover, the enriched biological processes were compared with those enriched in the GEO database of various types of human heart failure patients through Venn diagram analysis. Forty‐one biological processes overlapped between our mouse model and patients with congestive heart failure, 22 between our model and patients with ischaemic heart failure, and 11 between our model and patients with acute myocardial infarction (Figure 5f). The intersecting category among all the databases included extracellular matrix organization, leucocyte chemotaxis, vascular smooth muscle contraction, cardiomyocyte composition and contraction (Figure 5g). Thus, the biological processes in the heart failure model induced by left ventricular injection of oil were comparable to those in human ischaemic heart failure patients.

### 3.6 Treatment with nitroglycerin and sacubitril–valsartan in the mouse model with left ventricular injection of oil

**FIGURE 6:**
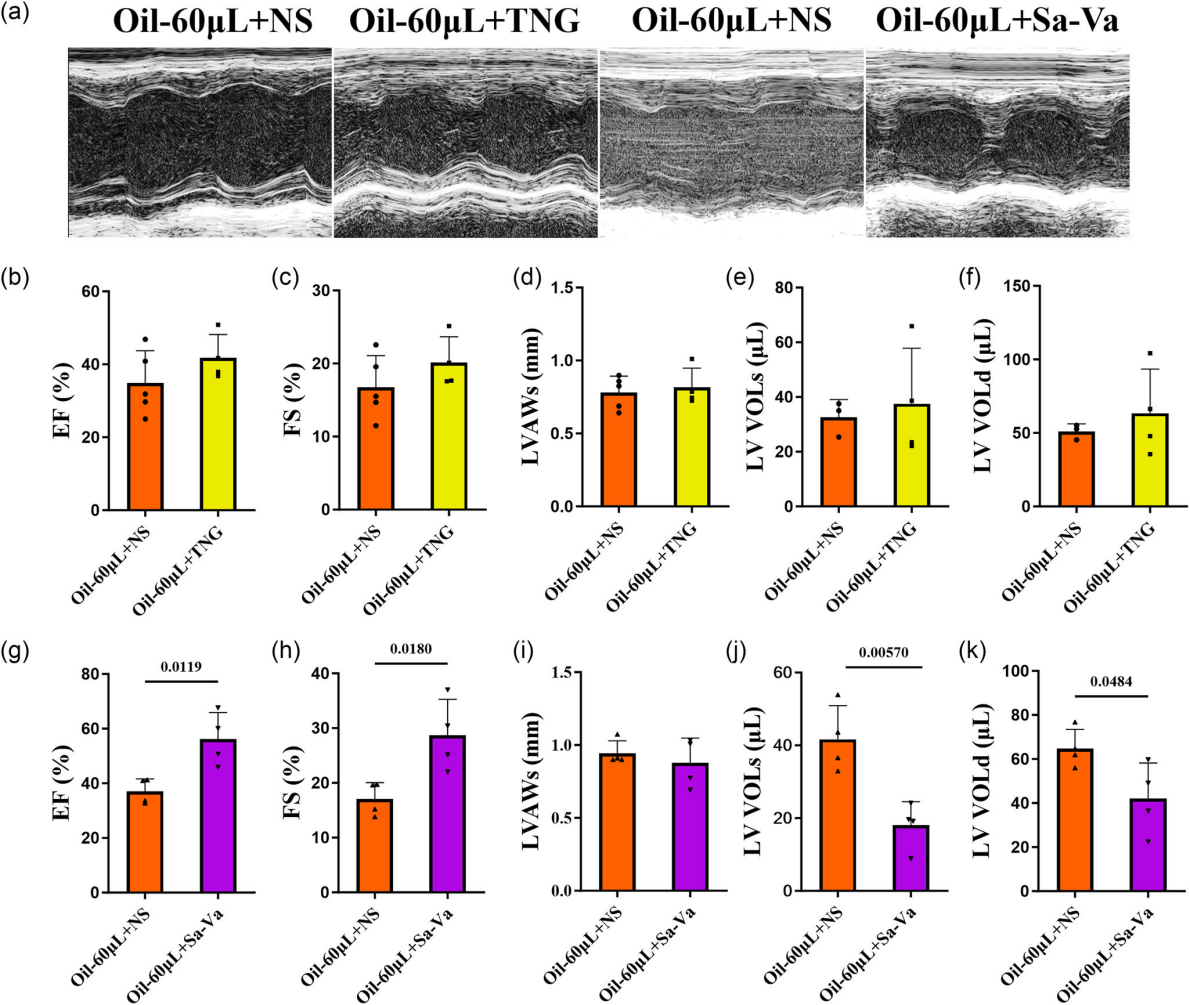
Heart function of the mouse model following 1 day of nitroglycerin or sacubitril–valsartan treatment. Two hours after oil injection, nitroglycerin (TNG; 25 mg/kg/day), sacubitril–valsartan (Sa‐Va; 60 mg/kg/day) or their vehicle control (normal saline, NS) was administered by intraperitoneal injection. (a) Echocardiography was performed on each group 1 day after drug treatment. (b–k) We analysed EF, FS, LVAWs, LV VOLs and LV VOLd after nitroglycerin (b–f) and sacubitril–valsartan (g–k) treatment. *n* = 4 or 5 mice per group; ^*^ *p *< 0.05 and ^**^ *p *< 0.01. Statistical analysis for (b–k) was performed using Student's *t*‐test. Results are presented as the mean ± SD.

**FIGURE 7:**
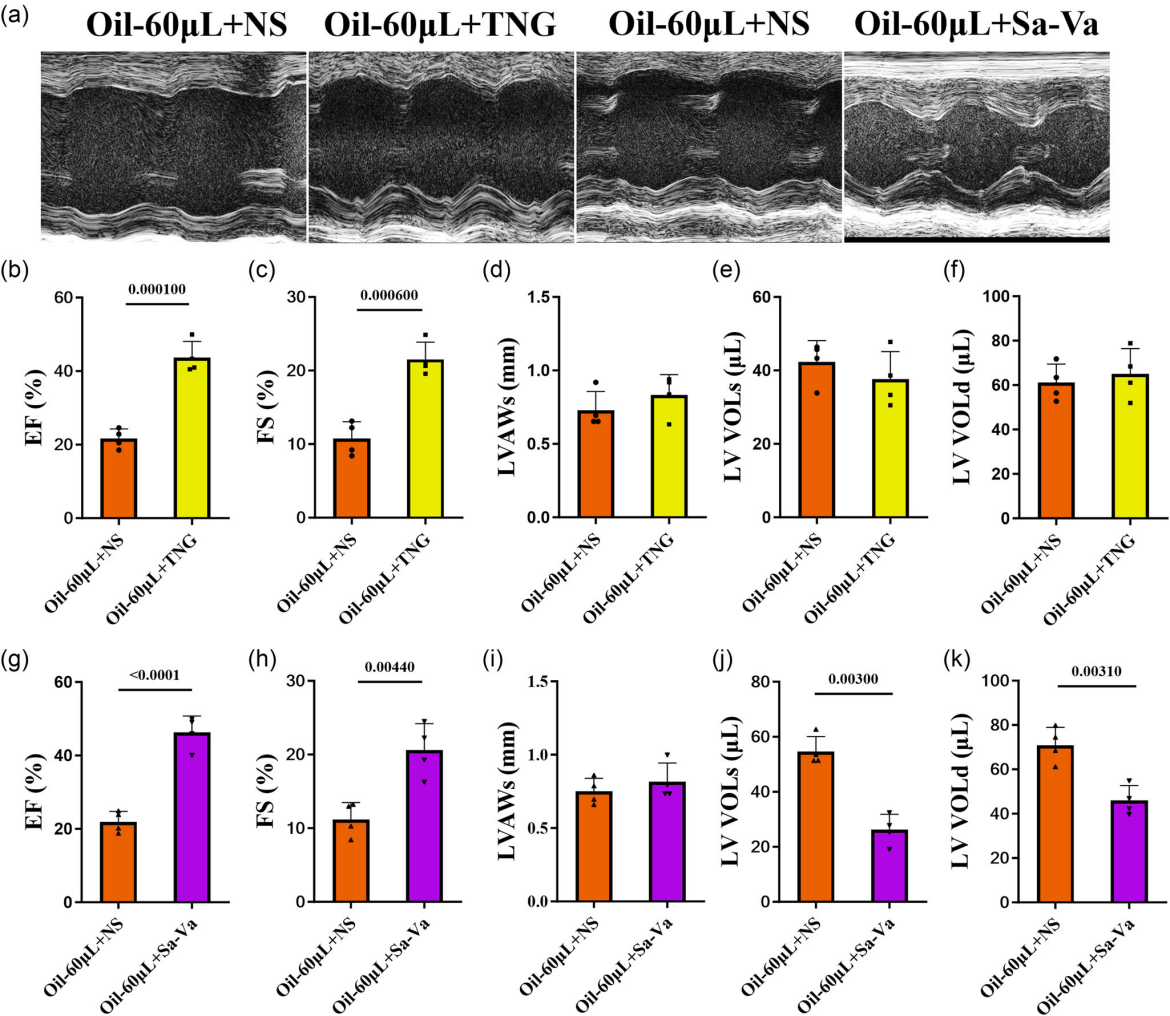
Heart function of the mouse model following 1 month of nitroglycerin or sacubitril–valsartan treatment. Two hours after oil injection, nitroglycerin (TNG; 25 mg/kg/day), sacubitril–valsartan (Sa‐Va; 60 mg/kg/day) or their vehicle control (normal saline; NS) was administered by intraperitoneal injection. (A) Echocardiography was performed on each group 1 month after drug treatment. (b–k) We analysed EF, FS, LVAWs, LV VOLs and LV VOLd after nitroglycerin (b–f) and sacubitril–valsartan (g–k) treatment. *n* = 4 or 5 mice per group; ^**^ *p *< 0.01, ^***^ *p *< 0.001 and ^****^ *p *< 0.0001. Statistical analysis for (b–k) was performed using Student's *t*‐test. Results are presented as the mean ± SD.

**FIGURE A6:**
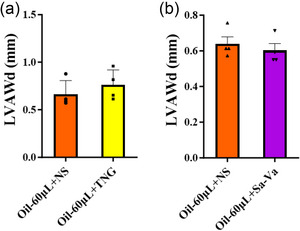
Diastolic left ventricular anterior wall thickness following 1 month of nitroglycerin or sacubitril–valsartan treatment. Two hours after 60 µL oil injection, nitroglycerin (TNG; 25 mg/kg/day), sacubitril–valsartan (Sa‐Va, 60 mg/kg/day) or their vehicle control (normal saline, NS) was administered by intraperitoneal injection, respectively. Echocardiography was performed on each group 1 month after drug treatment. Diastolic left ventricular anterior wall thickness (LVAWd) was analysed after nitroglycerin (a) or sacubitril–valsartan (b) treatment. *n* = 4 or 5 mice per group. Statistical analysis was performed using Student's *t*‐test. Results are presented as the mean ± SD.

Sacubitril–valsartan and nitroglycerin are widely used for the treatment of heart failure by reducing heart afterload and increasing heart blood supply, respectively. Thus, we evaluated the sensitivity to these drugs of the mouse model injected with oil. Compared with the 60 µL group, sacubitril–valsartan significantly enhanced the percentage of EF and FS and reduced left ventricular volumes during both systole and diastole after 1 day of treatment (Figure 6a, g–k, A5a). In contrast, nitroglycerin did not show a significant promotion in heart function after 1 day of treatment (Figure 6a, b–f, A5b). Notably, after 1 month of treatment with either nitroglycerin or sacubitril‐valsartan, the mouse model exhibited a sustained improvement in heart failure (Figures 7 and A6a, b). The data indicate that nitroglycerin and sacubitril–valsartan could improve heart function in mice with left ventricular injection of 60 µL of oil.

## 4 DISCUSSION

Although coronary microembolization and the resulting patchy ischaemic heart failure have garnered growing attention (Aggarwal et al., 2018), a suitable mouse model for studying this condition remains limited. In the present study, we successfully developed a mouse model of ischaemic heart failure induced by coronary microembolization through oil injection into the left ventricle. The model was characterized by immediate microvascular obstruction, cardiomyocyte necrosis, apoptosis and inflammatory infiltration, in addition to ongoing scarring of the heart tissue. RNA‐seq analysis revealed significant alterations in genes related to myocardial contraction, vasodilatation, myocardial fibrosis and chemotactic inflammation. Furthermore, treatment with nitroglycerin or sacubitril–valsartan was found to improve heart function effectively in the mouse model. Taken together, we used left ventricular oil injection to establish a mouse model of ischaemic heart failure induced by coronary microembolization, with high sensitivity to nitroglycerin and sacubitril–valsartan.

In our mouse model, the administration of oil resulted in an immediate obstruction of blood flow in numerous coronary microvessels, along with reactive hyperaemia in the epicardial coronary arteries. This indicates that left ventricular injection of oil effectively and mechanically induced obstruction of the coronary microvasculature. In patients, coronary microembolization caused by debris and thrombotic materials released from atherosclerotic plaques can lead to local necrosis, microinfarction and a pronounced inflammatory response, in addition to a sudden increase in serum levels of the myocardial injury marker CK‐MB (Heusch, 2022; Pernet et al., 2014; Schwartz et al., 2009). In animal models of microembolization, a rapid increase in inflammatory infiltration, necrosis, apoptosis and serum CK‐MB levels was also observed shortly after administration of microspheres or thrombotic particles (Cao et al., 2016; Dorge et al., 2002; Grimm et al., 1998; Grund et al., 1999; Saeed et al., 2013). In line with these findings, microembolization triggered an acute inflammatory reaction and an elevation of serum CK‐MB levels in our model. DEGs in inflammatory response and leucocyte chemotaxis were also enriched, with the neutrophil markers *S100a8*, *Csf3r*, *Cxcr2* and *Retnlg* being upregulated (Calcagno et al., 2021). The multiple small areas of myocardial necrosis and apoptosis indicated the presence of numerous microischaemic foci in the hearts in our model. These patches of ischaemia in different areas are likely to have decreased regional myocardial contractile function (Heusch, 2021, 2022), resulting in global acute left ventricular contractile dysfunction. Collectively, our mouse model replicated the acute pathological processes in microembolization. Additionally, the slight but not statistically significant increase in left ventricular EF and FS observed in the 40 µL group compared with the other oil‐treated groups might be attributed to individual variations in baseline heart function and the structure of the coronary arteries among the mice.

In patients, the rapid induction of local necrosis, microinfarction and inflammation caused by microembolization activates fibroblasts and inflammatory cascades, leading to further impairment of myocardial contractile function (Heusch et al., 2018). In our model, although the obstruction with oil had recovered 1 month after injection, heart function exhibited an irreversible deterioration, suggesting that the progressive damage in heart function was not attributable to the persistent obstruction, but to the initial injury to cardiomyocytes and their caspases, with possible contributions from ischaemia–reperfusion injury (Kalogeris et al., 2017).

Notably, both the decline in heart function and the development of fibrosis displayed a pattern that was dependent on the dose of oil administered. This dependence might stem from the initial dose‐dependent increase in inflammation and its signalling cascade, leading to the generation of reactive oxygen species, myofibrillar protein oxidation, decreased Ca^2+^ responsiveness and subsequent dose‐dependent impairment of the contractile ability of the myocardium (Canton et al., 2006; Dorge et al., 2002; Mao et al., 2019; Skyschally et al., 2008). Concurrently, dose‐dependent cardiomyocyte death and inflammatory responses triggered cardiac fibrosis, resulting in the significant and rapid regulation of genes involved in collagen formation, tissue remodelling and fibre organization. This would lead to increased ventricular stiffness, further compromising ventricular contraction and output (Murtha et al., 2017). As a result, heart function injury and cardiac fibrosis were also exacerbated in a dose‐dependent manner in our model.

Taken together, this indicates that our model can serve as a prolonged ischaemic heart failure model induced by microvascular obstruction, with the severity of heart failure being modulated by the volume of oil injected. In addition, the absence of apoptosis 1 month after injection aligns with established post‐infarction dynamics: apoptosis peaks within 48 h (Krijnen et al., 2002), with clearance occurring within 72 h in murine models (Bialik et al., 1997). Our RNA‐seq and RT‐PCR results revealed a biphasic *COL1A1* mRNA pattern (transient early suppression preceding later induction), reflecting progressive extracellular matrix remodelling and aligning with canonical fibrotic progression (Schumacher et al., 2022; Willems et al., 1994). The mechanisms driving this transient early suppression remain unclear and require further investigation.

Our mouse model, created through left ventricular oil injection, effectively mimics both the acute and prolonged pathological processes associated with microembolization and microembolization‐induced ischaemic heart failure. In comparison to microembolization models developed in sheep, dogs and pigs, mouse models are more cost‐effective and convenient, and they offer an easily modifiable platform for gene editing that facilitates more extensive research into the underlying pathogenesis. Notably, unlike the mouse microembolization model induced by left ventricular injection of microspheres (Cao et al., 2016), left ventricular injection of oil did not disrupt the morphological organization and blood perfusion of the brain, kidney and spleen in our model. This could be attributed to two potential factors. First, the injected volume of 60 µL in our model was less than one‐third of the total volume of the microspheres injected. Second, Takada et al. (2015) found that intravenous administration of emulsified oleic acid or linoleic acid (the major components of corn oil) did not result in fat emboli in the heart, lung, kidney and brain, in contrast to neutral fat isolated from white adipose tissue. In our model, after left ventricular injection, a portion of corn oil might be perfused directly into coronary arteries, while the remaining portion might be pumped into the aorta. Given the high‐frequency constriction of the left ventricle in mice, we infer that corn oil circulating into the aorta has undergone emulsification and is ultimately absorbed and metabolized by the liver (Vilaro & Llobera, 1988). Although we cannot control the distribution of oil precisely, we could regulate the severity of heart failure by adjusting the volume of oil injected, without affecting other peripheral organs. Thus, our mouse model of microembolization showcased high survival and ease of use, low cost and characteristic phenotypes. Nevertheless, a limitation of our model is the inert nature of oil. Although free fatty acids (including oleic acid and linoleic acid) are toxic to endothelial cells (Ghosh et al., 2017), corn oil, in contrast to microembolization induced by plaque erosion or rupture in patients, does not possess the ability to release vasoactive compounds, such as angiotensin II, nitric oxide, endothelin‐1 and prostacyclin, which are essential mediators for modulating vasoconstriction, vasodilatation and vasospasm (Smati et al., 2024).

Both sacubitril–valsartan and nitroglycerin significantly improved cardiac function in our mouse model, highlighting the high sensitivity of the model to these drugs. Neither drug restored heart contractility, but sacubitril–valsartan effectively reduced ventricular volume during both systole and diastole in our model, a benefit not observed with nitroglycerin. Sacubitril is a neprilysin inhibitor, and valsartan is an angiotensin receptor blocker. The neprilysin inhibitor induces vasodilatation, diuresis and natriuresis by inhibiting the hydrolytic activity of neprilysin on atrial natriuretic peptide, whereas angiotensin receptor blockers reduce cardiac afterload by selective inhibition of angiotensin II. Consequently, sacubitril–valsartan decreases blood resistance during cardiac ejection, alleviating heart congestion in our model (Gabriel Sayer, 2014; Hubers & Brown, 2016; Ksiazczyk & Lelonek, 2020). In contrast, nitroglycerin improves heart function by dilating coronary arteries and increasing oxygen supply to cardiomyocytes, but it does not affect cardiac afterload or peripheral resistance, resulting in no noticeable improvement in ventricular enlargement in our model (Elkayam et al., 2004).

## 5 CONCLUSION

In conclusion, we successfully developed a mouse model of ischaemic heart failure induced by coronary microembolization, characterized by ease of manipulation and time efficiency. This model might serve as a valuable tool for investigating the pathogenetic mechanisms and treatment of coronary microembolization‐induced ischaemic heart failure.

## AUTHOR CONTRIBUTIONS

Study design: Zhenwen Chen, Xiaoyan Du, Meng Guo and Lang Pei. Paraffin embedding: Lang Pei and Qiuyue Wu. Histological analysis: Lang Pei and Meng Guo. Immunohistochemical analysis: Lang Pei and Yusheng Hong. Echocardiographic analysis: Lang Pei and Yusheng Hong. Analysis of serum CK‐MB levels: Lang Pei and Changlong Li. RT‐PCR: Lang Pei and Xueyun Huo. RNA‐seq analysis: Lang Pei, Di Zhang, Meng Guo and Xiaoyan Du. GEO data analysis: Lang Pei, Meng Guo and Jianyi Lv. Manuscript writing: Meng Guo and Lang Pei. All authors have approved the final version of the manuscript and agree to be accountable for all aspects of the work in ensuring that questions related to the accuracy or integrity of any part of the work are appropriately investigated and resolved. All persons designated as authors qualify for authorship, and all those who qualify for authorship are listed.

## CONFLICT OF INTEREST

The authors declare that they do not have any competing financial interest.

## Supporting information



Supplementary Materials.

## Figures and Tables

**FIGURE A5:**
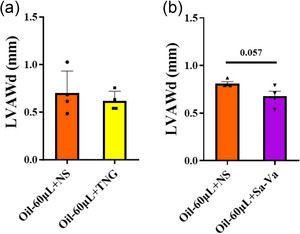
Diastolic left ventricular anterior wall thickness following 1 day of nitroglycerin or sacubitril–valsartan treatment. Two hours after 60 µL oil injection, nitroglycerin (TNG; 25 mg/kg/day), sacubitril–valsartan (Sa‐Va; 60 mg/kg/day) or their vehicle control (normal saline, NS) was administered by intraperitoneal injection., Echocardiography was performed on each group 1 day after drug treatment. Diastolic left ventricular anterior wall thickness (LVAWd) was analysed after nitroglycerin (a) or sacubitril–valsartan (b) treatment. *n* = 4 or 5 mice per group. Statistical analysis was performed using Student's *t*‐test. Results are presented as the mean ± SD.

## Data Availability

The RNA‐seq data were uploaded as Supporting Information.

## Appendix

**TABLE A1:** Primers used in our study.

Gene	Forward	Reverse
*MYH7*	5′‐AGTCCCAGGTCAACAAGCTG‐3′	5′‐TTCCACCTAAAGGGCTGTTG‐3′
*BNP*	5′‐GGTCCAGCAGAGACCTCAAAAT‐3′	5′‐CAACAACTTCAGTGCGTTACAG‐3′
*ACTB*	5′‐CCGGCAAAAATCACACGCAG‐3′	5′‐AGTCCCAGGTCAACAAGCTG‐3′
*TIMP1*	5′‐ACGAGACCACCTTATACCAG‐3′	5′‐GCTTTCCATGACTGGGGTGT‐3′
*COL1A1*	5′‐CGCCATCAAGGTCTACTGC‐3′	5′‐ACGGGAATCCATCGGTCA‐3′
